# Risk of subsequent invasive breast carcinoma after *in situ* breast carcinoma in a population covered by national mammographic screening

**DOI:** 10.1038/sj.bjc.6602250

**Published:** 2004-11-30

**Authors:** R Rawal, J Lorenzo Bermejo, K Hemminki

**Affiliations:** 1 German Cancer Research Center, Division of Molecular Genetic Epidemiology, Im Neuenheimer Feld 580, Heidelberg D-69120, Germany; 2 Department of Biosciences at Novum, Karolinska Institute, Huddinge, Sweden

**Keywords:** DCIS, LCIS, *in situ* breast cancer, invasive breast cancer

## Abstract

Sweden was the first country to establish a nationwide breast cancer screening service. We used the Swedish Family-Cancer Database to evaluate the risk of invasive carcinoma after *in situ* carcinoma of the breast. Risk estimates for contralateral and ipsilateral invasive malignancies following age and histology specific *in situ* breast carcinomas were calculated using Poisson's regression analysis. The agreement between concordant and discordant morphologies of invasive and *in situ* breast cancer was measured using the kappa statistic. Women with *in situ* breast cancer showed a relative risk of 2.03 for contralateral and 3.94 for ipsilateral invasive breast cancer. The risk was higher for *in situ* carcinomas diagnosed before the age of 50 years and after lobular *in situ* breast cancers. A comparison of the risks during the past decades suggested that the risk of ipsilateral breast cancer has increased in Sweden but that of contralateral breast cancer has remained unchanged. *In situ* and the subsequent invasive breast cancers did not seem to share their morphologies.

The most common morphologies of carcinoma *in situ* of the breast are ductal (DCIS) and lobular carcinoma (LCIS). In Sweden, the incidence of DCIS has increased since the introduction of mammographic screening, representing now 15–20% of all breast malignancies, compared with 5% of the cases in prescreening era (Pinder and Ellis, 2003). The incidence of LCIS has also increased, but appreciably slower than that of DCIS (Ernster *et al*, 1996). In addition to the different incidence patterns, mostly due to mammographic characteristics, DCIS is regarded as a local precursor of invasiveness, while LCIS is merely considered a general risk factor for developing invasive carcinoma, including a contralateral malignancy (Claus *et al*, 2003; Page, 2004). The estimated risks of invasive breast cancer after *in situ* carcinoma are heterogeneous in the literature: DCIS has been associated with a four- to 10-fold risk increment; the estimates of the relative risk (RR) after LCIS vary from 2 to 11 (Page *et al*, 2000; Warnberg *et al*, 2000).

Sweden has been the first country that implemented a nation-wide mammographic screening. The first service was started in year 1986 and it was extended to a complete national coverage by year 1997 (Olsson *et al*, 2000). One consequence of screening has been an earlier detection of breast cancer (Zahl *et al*, 2004). The conservative breast surgery may have also influenced the clinical course and prognosis of breast cancer (Kestin *et al*, 2000). The aim of the present study was to evaluate the risk of invasive breast carcinoma after *in situ* carcinoma in Sweden. The estimation was based on cancers diagnosed between 1993 and 2000, thus taking into account the possible impact of screening and recent advances in management of *in situ* breast cancers. Risk estimates were derived for contralateral and ipsilateral invasive malignancies following age- and histology-specific *in situ* breast carcinomas. The change in the risk of invasive carcinoma after *in situ* breast cancer during the last 20 years in Sweden was also explored.

## MATERIALS AND METHODS

**Figure 1:**
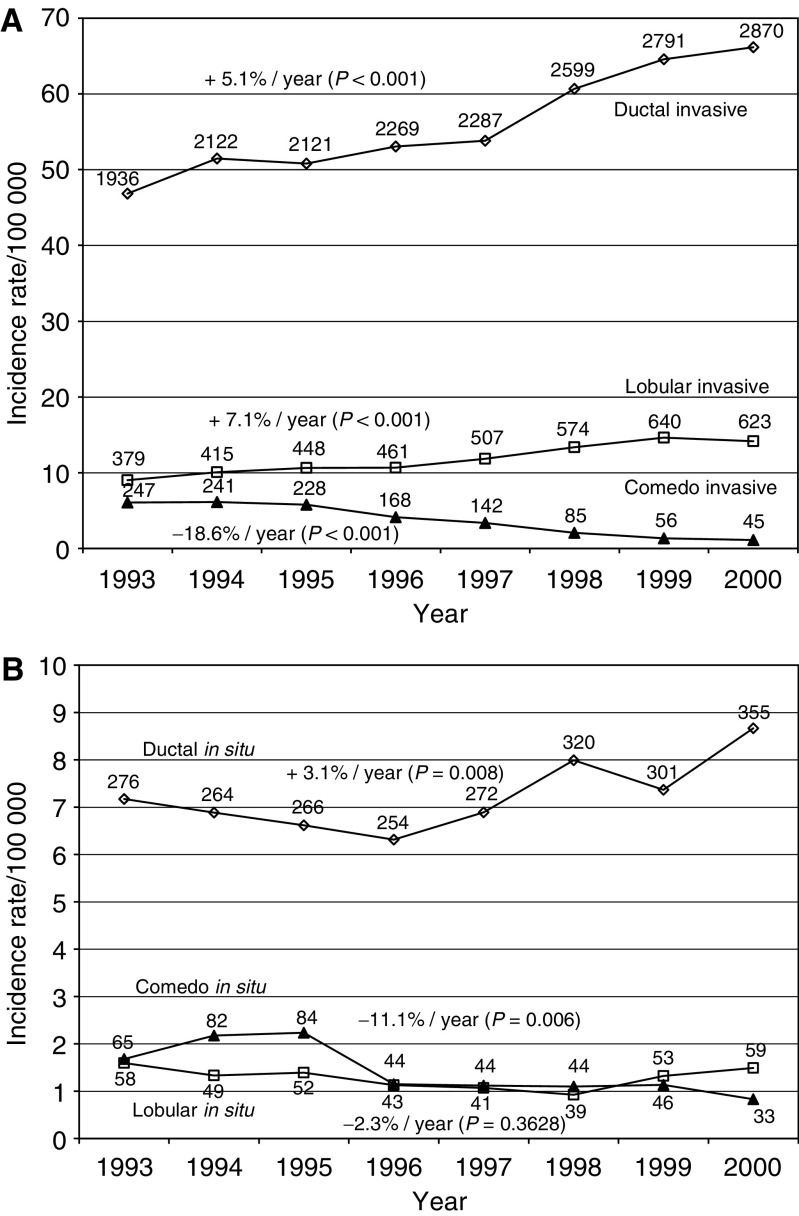
Trends in incidence rates of invasive (**A**) and *in situ* breast cancer (**B**) according to histologic subtype, based on the Swedish Family-Cancer Database. Incidence rates are adjusted for age (European standard). The mean annual increase or decrease is presented at the curve. The numbers of observed cases are presented for each year.

The Swedish Family-Cancer Database was created in the mid-1990s by linking census information, death notifications and the administrative family register to the Swedish Cancer Registry at Statistics Sweden (Hemminki, 2001; Hemminki *et al*, 2001). The Database was updated at the end of 2002 to include more than 10 million individuals. The present analysis considered diagnoses made between 1993 and 2000 and included 3802 *in situ* breast and 35 480 invasive breast cancers in a population of five million women. The Swedish Cancer Registry is based on compulsory reports of individual cases provided by clinicians/pathologists or cytologists and is considered to have almost 100% completeness (The National Board of Health and Welfare Stockholm, 2002). The incidence of cancer in the Database is similar to the incidence in the Cancer Registry (Hemminki *et al*, 2001; Plna and Hemminki, 2001). Four-digit diagnostic codes from the seventh revision of the International Classification of Diseases (ICD-7) and subsequent ICD classifications are available. In the current study, carcinoma of breast was indicated by ICD-7 code 170. Histology was recorded according to the Systemized Nomenclature of Medicine (SNOMED). Invasive breast cancers were classified as: ductal carcinoma (SNOMED code 85003), lobular carcinoma (85203) and comedo carcinoma (85013). *In situ* breast cancers were also classified in three categories: ductal carcinoma (85002), lobular carcinoma (85202) and comedo carcinoma (85012). Breast cancer is often of mixed morphology at presentation and only the main type was recorded in the Swedish Cancer Registry (Harris *et al*, 1993). Invasive breast cancers diagnosed after *in situ* malignancies in the other breast were classified as contralateral, but as ipsilateral if they occurred in the same breast. Only invasive cancers diagnosed at least 1 month after diagnosis of the *in situ* breast carcinoma were included in the present study. Incidence rates of histology-specific breast cancers were standardised for age according to the European population. Variations in incidence rates from 1993 to 2000 were studied by log linear regression analyses based on the maximum-likelihood method (Figure 1).

Follow-up began at the age of 21 years (earliest age at diagnosis of invasive breast cancer), the date of immigration, the date of diagnosis of *in situ* breast cancer or 1 January 1993, whichever occurred last. Follow-up ended at diagnosis of the first primary malignancy, death date, emigration date or 31 December 2000, whichever occurred first. Person-years and cases of invasive breast cancer were counted and grouped by age, family history, age at first birth and the presence or absence of *in situ* breast cancer. A Poisson regression analysis was applied to the data using the Genmod procedure of the SAS program (SAS/STAT® User's guide, 1999). The results are shown as an RR, with 95% confidence limits (95% CI). Risk estimates were also calculated for the periods 1981–1990, 1991–1995 and 1996–2000 in order to investigate possible changes during the last 20 years in Sweden. Differences in the time of follow-up and age of the women among the three periods were taken into account by indirect standardisation before Poisson's regression, using the period 1996–2000 as reference.

The kappa statistic was used as a measure of agreement between concordant and discordant morphologies of invasive and *in situ* breast cancer. Kappa takes values between −1 and 1, where 0 indicates no determination and −1 or 1 would indicate that the morphology of the invasive carcinoma is completely determined by the histology of the prior *in situ* carcinoma. Values of the kappa statistic between 0.40 and 0.60 would suggest a moderate *concordance*.

## RESULTS

The incidence rate of invasive ductal cancer increased in the period 1993–2000 from 46.8 to 66.1/100 000 (+5.1% per year) and invasive lobular cancer showed an increase from 9.0 to 14.2/100 000 (+7.1% per year). A striking decrease was found for the comedo histology, with incidence rates from 6.1 to 1.1/100 000 (−18.6% per year). *In situ* ductal cancer increased from 7.2 to 8.7/100 000 (+3.1% per year) and, interestingly, the incidence of *in situ* lobular cancer remained constant (1.6/100 000). *In situ* comedo cancer showed a decreasing incidence rate from 1.7 to 0.8/100 000 (−11.1% per year).

The results of the Poisson regression analysis for contralateral breast cancer are presented in Table 1. The diagnosis of *in situ* breast carcinoma resulted in a two-fold increase in the risk of invasive cancer in the contralateral breast; the increase was particularly high if the *in situ* breast lesion was diagnosed before the age of 50 years. The risk increased with the time after diagnosis of the *in situ* carcinoma, although the increment was not statistically significant. The lobular histology of *in situ* breast carcinoma was associated with the highest risk for contralateral invasive breast cancer. The diagnosis of *in situ* carcinoma resulted in an RR of 3.94 for invasive ipsilateral cancer (Table 2). Comedo *in situ* carcinoma was associated with the highest risk of ipsilateral invasive breast cancer (RR=5.02).

**Figure 2:**
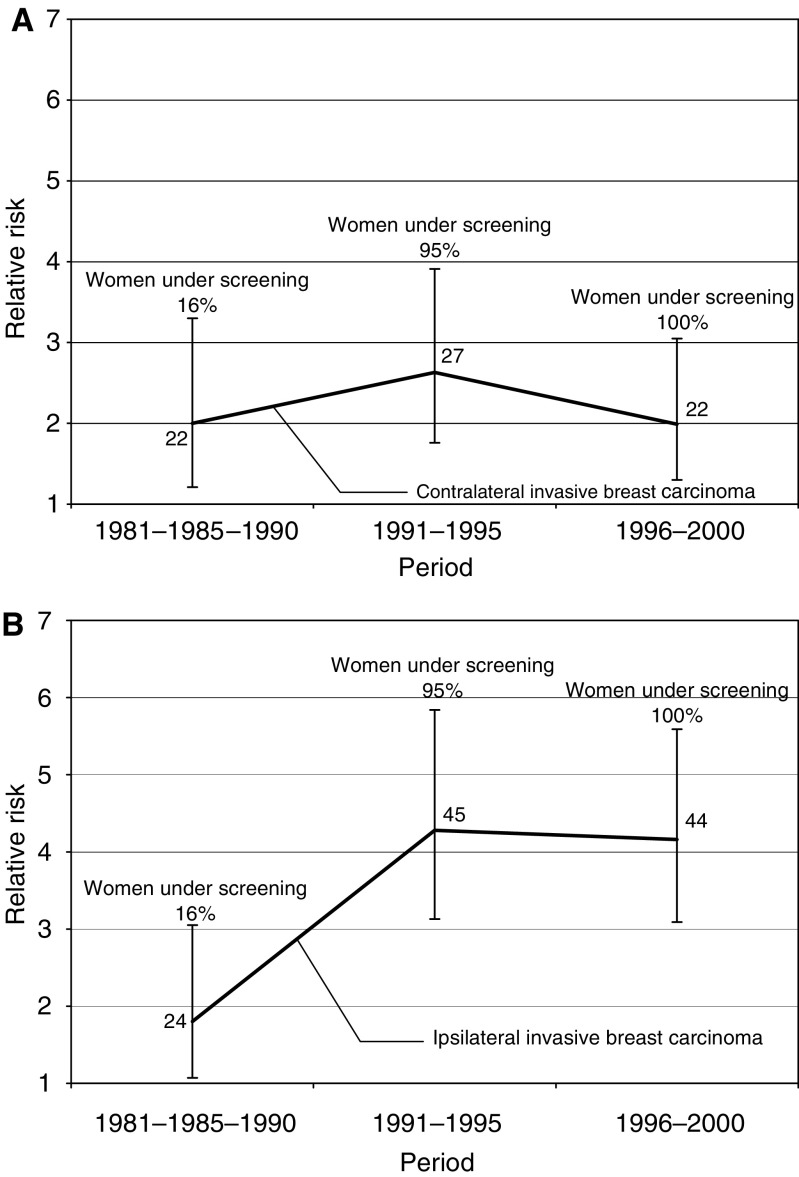
Relative risk of contralateral (**A**) and ipsilateral (**B**) invasive breast carcinoma subsequent to *in situ* breast carcinoma for different periods.

The risks of contralateral invasive cancer after *in situ* breast cancer slightly increased during the period 1991–1995 and it decreased thereafter, but were not statistically significant (Figure 2A). In contrast, the risks of ipsilateral invasive breast cancer after *in situ* breast cancer in 1991–1995 and 1996–2000 were two times higher than in 1981–1990 (Figure 2B); the risk differences were statistically significant. The increase in the risk of ipsilateral breast cancer was mostly associated with *in situ* carcinomas diagnosed at ages 50–60 years (results not shown).

Table 3 shows kappa measures of agreement between concordant *in situ* and invasive morphologies. Kappa values for all concordant and discordant histologies (results not shown for discordant histologies) were lower than 0.40, thus suggesting that the histology of the invasive cancers was not determined by the morphology of the preceding *in situ* carcinoma.

## DISCUSSION

The treatment for the first breast cancer, the intense medical follow-up and the self-observation of the patient complicate the epidemiology of second breast cancers. Moreover, two breasts are at risk for the first cancer, but only one of them is at risk for ipsilateral or for contralateral breast cancer. Treatment, follow-up and definition differences have probably contributed to the incongruent results for invasive breast cancer after *in situ* carcinoma in the literature (Chen *et al*, 1999). The RRs shown in the present study were calculated after dividing the second breast cancers into contralateral and ipsilateral breast cancers.

All *in situ* breast carcinomas are premalignant, being possible precursors to invasive disease capable of metastasis (Dupont and Page, 1985; Page *et al*, 1985, 2000; Page and Dupont, 1990; London *et al*, 1992). The potential for progression to invasive cancer has been measured in earlier studies (Franceschi *et al*, 1998; Ottesen *et al*, 2000; Crocetti *et al*, 2001; Warnberg *et al*, 2001). However, the number of cases analysed previously was small. One study considered breast cancers in the period 1980–1992, that is, before and during the establishment of screening services in Sweden (Warnberg *et al*, 1999). Earlier diagnosis due to screening and recent advances in the treatment of *in situ* carcinoma have resulted in better survival rates for patients and a higher incidence for invasive carcinoma. An advantage of the present study was the adjustment for age, family history and parity in the Poisson regression analyses. Unfortunately, information on possible confounders such as treatment received, use of contraceptives and stage of the cancer or tumour size was not available.

The increased incidence of invasive ductal and lobular breast cancers found in the present study was similar to previous reports (Levi *et al*, 1997; Li *et al*, 2003; Verkooijen *et al*, 2003). The reduction in the incidence of comedo breast cancer is probably attributable to modifications in pathological classification criteria. The incidence of DCIS increased in the period 1993–2000, which was to a small extent due to the reclassification of comedo *in situ* carcinomas as DCIS. In contrast, the incidence of LCIS did not change. Earlier reports have shown increases in the incidence of DCIS and LCIS (Simon *et al*, 1993; Choi *et al*, 1996; Levi *et al*, 1997). The different incidence patterns found for DCIS and LCIS in the present study may be partly attributable to the low calcification of lobular tumours, which hampers their detection by mammography.

*In situ* breast carcinoma diagnosed before the age of 50 years was associated with the highest risk of invasive cancer, the difference from other age groups was even statistically significant for ipsilateral breast cancer. In patients with mammographically detected ductal carcinoma *in situ*, treated with breast-conserving therapy, young patient age has been reported to be a risk factor for local recurrence (Kestin *et al*, 2000). Our results were similar to earlier studies, which have shown higher risks for invasive breast cancer in women with *in situ* cancer diagnosed at young ages (Franceschi *et al*, 1998; Crocetti *et al*, 2001; Warnberg *et al*, 2001; Claus *et al*, 2003). The risk of invasive breast cancer increased with the time after diagnosis of *in situ* carcinoma, but the number of cases analysed was small and the trend was not statistically significant. Our data agree with earlier results, that the highest risk of invasive cancer is reached after 42 to 60 months after diagnosis of *in situ* lesions. (Franceschi *et al*, 1998; Ottesen *et al*, 2000; Warnberg *et al*, 2000, 2001; Crocetti *et al*, 2001).

The risk of invasive cancer in the contralateral and ipsilateral breasts was higher after lobular than after ductal carcinoma *in situ*. Our results therefore disagree with the report suggesting that this risk was higher after DCIS than after LCIS (Warnberg *et al*, 2000). A higher incidence of DCIS due to screening may have diluted the risk of the subsequent invasive breast cancer, whereas the incidence of LCIS and its corresponding risk seem to have been only slightly modified.

The estimated risk of ipsilateral invasive cancer after *in situ* lesions was almost two times higher than the corresponding risk of contralateral breast cancer. This risk difference was only found after 1991, and was statistically significant for the period 1996–2000. Breast-conserving surgery became the treatment of choice for early breast cancer in Sweden during the 1980s, especially in areas with population-based screening (Fredriksson *et al*, 2001). The proportion of women receiving conservative surgery increased from 7% in 1980 to 51% in 1996 (Lindqvist *et al*, 2002). Inadequately treated *in situ* breast carcinoma, for example, insufficient margin control, may have contributed to the observed risk increase (Yau *et al*, 2002; Fredriksson *et al*, 2003; Kerlikowske *et al*, 2003; Khan and Newman, 2004).

The suggestion of previous studies that *in situ* and subsequent invasive cancers share morphological features (Habel *et al*, 1997; Franceschi *et al*, 1998) could not be confirmed in the present study. However, we found the highest concordance for the lobular histology, in agreement with earlier studies, which showed that LCIS is often followed by lobular invasive breast cancer (Lishman and Lakhani, 1999; Fisher *et al*, 2004). The present study is congruent with earlier analyses on the genetic determination of the morphology of invasive breast cancer based on the Swedish Family-Cancer Database (Hemminki and Granstrom, 2002).

We conclude that the incidence of *in situ* carcinoma of the breast has increased in the last decade and that the introduction of screening and new treatments in Sweden seems to have modified the pattern of risk of invasive cancer after *in situ* carcinoma. The risk of invasive cancer in the ipsilateral breast has increased. Women with LCIS are at higher risk of invasive breast cancer than women with DCIS. The risks estimated in this study may help in clinical counselling.

## Figures and Tables

**Table 1:** Contralateral invasive breast cancer subsequent to *in situ* carcinoma of breast analysed in Poisson's regression model

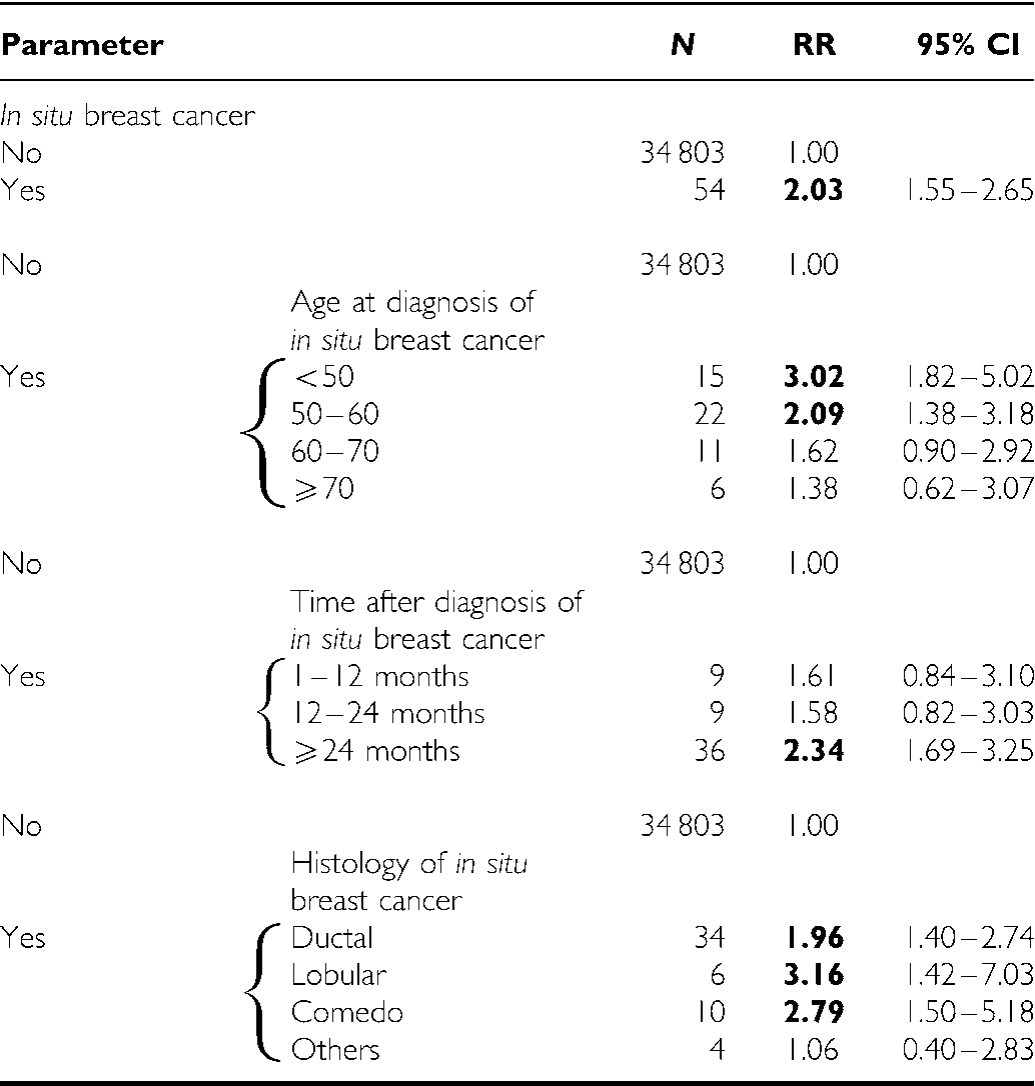

RR=relative risk; CI=confidence interval.

Data were additionally adjusted for age, family history, parity and age at first birth.

Bold=the RR was statistically higher than 1.00.

**Table 2:** Ipsilateral invasive breast cancer subsequent to *in situ* carcinoma of breast analysed in Poisson's regression model

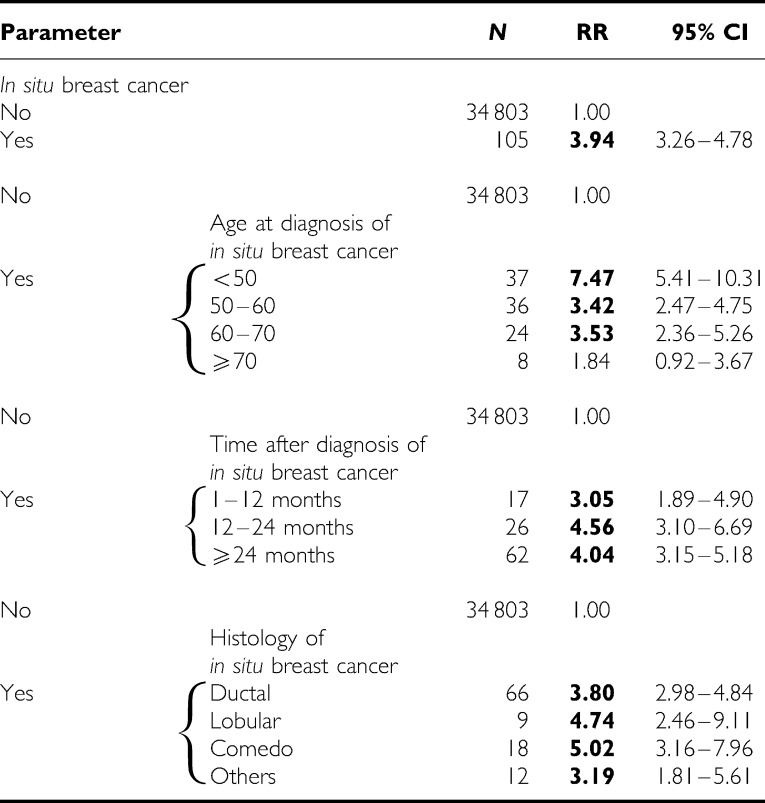

RR=relative risk; CI=confidence interval.

Data were additionally adjusted for age, family history, parity and age at first birth.

Bold=the RR was statistically higher than 1.00.

**Table 3:** Kappa measures of agreement between concordant *in situ* and invasive morphology

	**Kappa**					
	***N***	**All morphologies**	***N***	**Ductal (Ductal+comedo)**	***N***	**Lobular**
						
All *in situ* followed by invasive	140	0.19	62	0.21	5	0.34
Invasive in contralateral breast	54	0.14	24	0.16	2	0.29
Invasive in ipsilateral breast	105	0.18	47	0.18	5	0.36
